# The Challenges and Opportunities of Sustaining Academia-Sponsored Community Service Programs for Latinx Youth During the COVID-19 Pandemic

**DOI:** 10.1177/1538192720980294

**Published:** 2021-07

**Authors:** Cheryne M. Kim, Brittany R. Silverman, Claudio Cortes

**Affiliations:** 1 Oakland University William Beaumont School of Medicine, Rochester, MI, USA

**Keywords:** community partnership, community service, COVID-19, Hispanic, Latino, Latinx, medical education, medical students, pandemic, service, underserved populations

## Abstract

The COVID-19 pandemic has widely affected existing academia-sponsored community service initiatives. Little is known about the strategies to sustain these initiatives during a public health crisis and the potential effects on community well-being and education. In this case study, we describe the impact of the pandemic on service partnerships between our medical school and the Latinx community, discuss the challenges and opportunities of transitioning to a virtual community service model, and offer solutions and considerations.

## Resumen

La pandemia de COVID-19 ha afectado ampliamente las iniciativas de servicio comunitario existentes y apoyadas por la academia. Poco se sabe acerca de las estrategias para sostener esas iniciativas durante una crisis de salud pública, y los efectos potenciales en el bienestar y educación comunitarios. En este estudio de caso, describimos el impacto de la pandemia en alianzas de servicio entre nuestra escuela de medicina y la comunidad latinx, discutimos los retos y las oportunidades en la transición a una comunidad modelo de servicio virtual, y ofrecemos soluciones y consideraciones.

## Background

Academia-sponsored community service, especially in undergraduate and professional programs (e.g., medicine, dentistry, nursing), is uniquely positioned at the intersection of educating students in higher education and supporting the needs of the community. From an educator’s perspective, community service has demonstrated positive outcomes in clarifying career goals (Yao, 2008), civic responsibility (Astin & Sax, 1998), and self-efficacy (Vogelgesang & Astin, 2000) for students in higher education. It has also been shown to have a favorable impact on students’ understanding of social issues, personal insight, and cognitive development (Yorio & Ye, 2012).

The role of community service is particularly emphasized within medical education. The Liaison Committee on Medical Education (LCME), an accrediting body for medical schools in the U.S. and Canada, recognizes the benefits of community service in fulfilling medical curriculum requirements (LCME, 2018). One of its criteria includes creating opportunities to respond to the health demands of an increasingly diverse patient population and to learn the importance of cultural competence, gender and cultural biases, healthcare disparities, and professionalism. In fact, the LCME singles out community service as one of eight curricular objectives, thereby holding medical schools accountable for supporting medical student participation in service-learning and community service activities. Beyond the advantages highlighted by the LCME, community service has also been associated with enhanced medical student empathy (Brazeau et al., 2011) and improved performance outcomes in medical school academics and residency (Blue et al., 2006).

On a larger scale, community service has considerable potential in supporting the needs of historically underserved populations. Significant racial and ethnic disparities continue to exist within U.S. poverty rates, especially among children. With one in four children in the U.S. identifying as Hispanic/Latino, the Latinx youth population experiences disproportionately high rates of poverty (Turner et al., 2015). As of 2018, 26% of Hispanic/Latino children live in poverty, which is more than twice the rate of poverty among non-Hispanic white children (The Annie E. Casey Foundation Kids Count Data Center, 2019). This population is also less likely to attain higher education and academic success, in part due to a multitude of resource inequalities. Some of these inequalities include insufficient school funding, lack of culturally competent staff, and poorly developed or nonexistent bilingual and “English as a Second Language” programs (Arbelo Marrero, 2016). In an endeavor to address these deeply embedded issues, community service initiatives have emerged and taken various forms, such as tutoring and mentoring programs that partner at-risk Latinx youth with undergraduate or graduate students. For example, Coller and Kuo (2014) described a mentorship program for Latinx children in Los Angeles, California that aims to foster academic success, self-worth, and healthy relationships with others and diminish the potential for substance use and violence. There are also health promotion programs for Latinx youth on topics of nutrition and physical activity education (Arlinghaus et al., 2017), sexually transmitted disease prevention (Kelly et al., 2006), and wellness and empowerment (Cavazos Vela et al., 2019).

Unfortunately, the detrimental effects of the COVID-19 pandemic have further exacerbated injustices and strained community service efforts. According to the Centers for Disease Control and Prevention (2020a), COVID-19 has disproportionately affected racial and ethnic minority groups in both incidence and mortality, in large part due to systemic inequities in pre-existing health conditions, living conditions, occupational exposures, access to healthcare, etc. African-American and Hispanic/Latino individuals comprise 13.4% and 18.5% of the U.S. population respectively and yet account for 19.8% and 31.1% of COVID-19 cases as of August 18, 2020 (Centers for Disease Control and Prevention, 2020b; United States Census Bureau, 2019). Additionally, Hispanic/Latino individuals have also been disproportionately affected financially (Lopez et al., 2020). As of April 2020, 61% of Hispanic/Latino adults (compared to 38% of non-Hispanic/Latino white adults) have lost their jobs or are receiving reduced income, and 70% of Hispanic/Latino adults (compared to 47% of non-Hispanic/Latino white adults) did not have financial savings to cover 3 months of expenses (Lopez et al., 2020). The short-term effects of COVID-19 on minorities have been charted from multiple angles, but its long-term impact, especially on the most vulnerable groups, is still largely unknown.

One of the most vulnerable subsets of this underserved population has been school-age children. With the closure of nearly all schools across the U.S. and the decision to leave remote learning plans (or lack thereof) to the discretion of individual school districts (Potter, 2020), the COVID-19 pandemic could impose severe consequences on students from low-income households. The summer holiday is already associated with loss in academic achievement (Cooper et al., 1996) and setbacks in mental wellness (Morgan et al., 2019) for children of low socioeconomic status. Compounded with the unforeseen effects of COVID-19, premature school closures and unequal remote learning experiences (Potter, 2020) could imitate a prolonged summer holiday and exacerbate the aforementioned outcome disparities in K-12 education and psychological health (Van Lancker & Parolin, 2020). The disruption of the U.S. education system now heightens the need for summer enrichment programs geared toward economically and systemically disadvantaged students. Such interventions could be helpful in reducing inequalities, bridging the widening summer learning gap (McCombs et al., 2011), and improving mental health (Hutton & Nalamada, 2020).

In addition to heavy strain on certain minority populations, COVID-19 has placed challenges and restrictions on many community service efforts geared toward them. Although medical students across the nation have created student-service chapters and coordinated new community service opportunities (Mangan, 2020), the pandemic has disrupted existing medical school-led community service initiatives to various degrees. Many hospital volunteer programs have been suspended to curb the spread of the disease and protect vulnerable groups (Baker, 2020), and likely many medical schools and community organizations have been experiencing financial burdens to support their volunteer programs. Unfortunately, research has yet to capture and publish the full scope of the short- and long-term consequences of these disruptions and suspensions. Thus, amidst the number of new service initiatives created to help communities during a pandemic, it is equally imperative to nurture and sustain existing service programs and partnerships that support vulnerable populations.

To do this, service programs are likely having similar conversations about how to best adapt and operate in a socially distant world. In particular, for service programs centered around education and learning, conversations may, in part, be driven by existing literature on virtual learning frameworks (Kebritchi et al., 2017) and effective online teaching (Rose, 2018). Not only does the literature inadvertently offer solutions that abide by social distancing guidelines, but it also acknowledges that face-to-face teaching competencies can transfer to virtual contexts (Roddy et al., 2017). As a result, online programming has become especially relevant during this public health crisis.

Here, we describe the challenges and opportunities of sustaining an established community service partnership and program between Oakland University William Beaumont School of Medicine (OUWB) and the surrounding Latinx community. We also offer possible solutions that could help other undergraduate and professional institutions to develop similar virtual community service models.

## Challenges and Opportunities

For many years prior to the COVID-19 pandemic, our medical school has spearheaded several programs for local Latinx youth, including a summer anatomy program. These programs were made possible through a partnership with Hispanic Newcomer Outreach of Catholic Charities of Southeast Michigan (CCSEM-HNO), an organization that provides a variety of services such as occupational assistance, educational classes, and legal aid for Latinx families living in Pontiac, Michigan. Although the Hispanic/Latino population in Michigan is low (5.3%) when compared to other states such as California (39.4%), Texas (39.7%), and New Mexico (49.6%), an increasing number of Hispanic/Latino individuals are concentrated in resource-limited regions of Michigan (United States Census Bureau, 2019). For instance, with respect to our program, 19.6% of the population in Pontiac, Michigan is Hispanic/Latino (United States Census Bureau, 2019).

Beginning in March 2020, the COVID-19 pandemic put these programs at risk for cancellation, due to their reliance on interactive activities with face-to-face instruction. In fact, one was cancelled, due to limited human resources and its inherently didactic-heavy design. However, two programs were able to successfully transition to an online platform. Several common challenges and opportunities arose to move these two programs online, and we will focus on one of them, the summer anatomy program, to describe the process of sustaining an existing community service program during a pandemic.

The OUWB-HNO Summer Anatomy Program is a 3-week program for Latinx children (ages 7–13) living in or near Pontiac, Michigan. Around five to six medical student volunteers teach Latinx youth about wellness and health by providing exposure to various organ systems through the use of didactic lectures, interactive games, and projects. The program offers educational enrichment by fostering curiosity, excitement for learning, and interest in science, technology, engineering, and mathematics (STEM).

The key factors essential to transitioning to a virtual, sustainable program, from which most of the challenges and opportunities stemmed, included the following: (1) the strength of the institutional partnership, (2) the leadership structure, (3) the structural logistics, and (4) the design of the educational sessions.

### Strength of the Institutional Partnership

We understand that an important element in the sustainability of a community service program is the strength of the partnership between the academic institution (e.g., administration, faculty, students) and the community (Blank, 2015). Moreover, we acknowledge that a combination of unique factors may have helped to solidify the 4 year-long partnership between OUWB and CCSEM-HNO (Peters et al., 2020). Because the program is positioned within the local Latinx community, the interpersonal relationships and sociocultural exchanges may have been strengthened through nuances, such as language concordance among key members of the leadership team (community coordinator and medical school faculty advisor), the medical school’s curricular emphasis on cultural competence, and the involvement of the Latino Medical Student Association.

**Figure 1.**
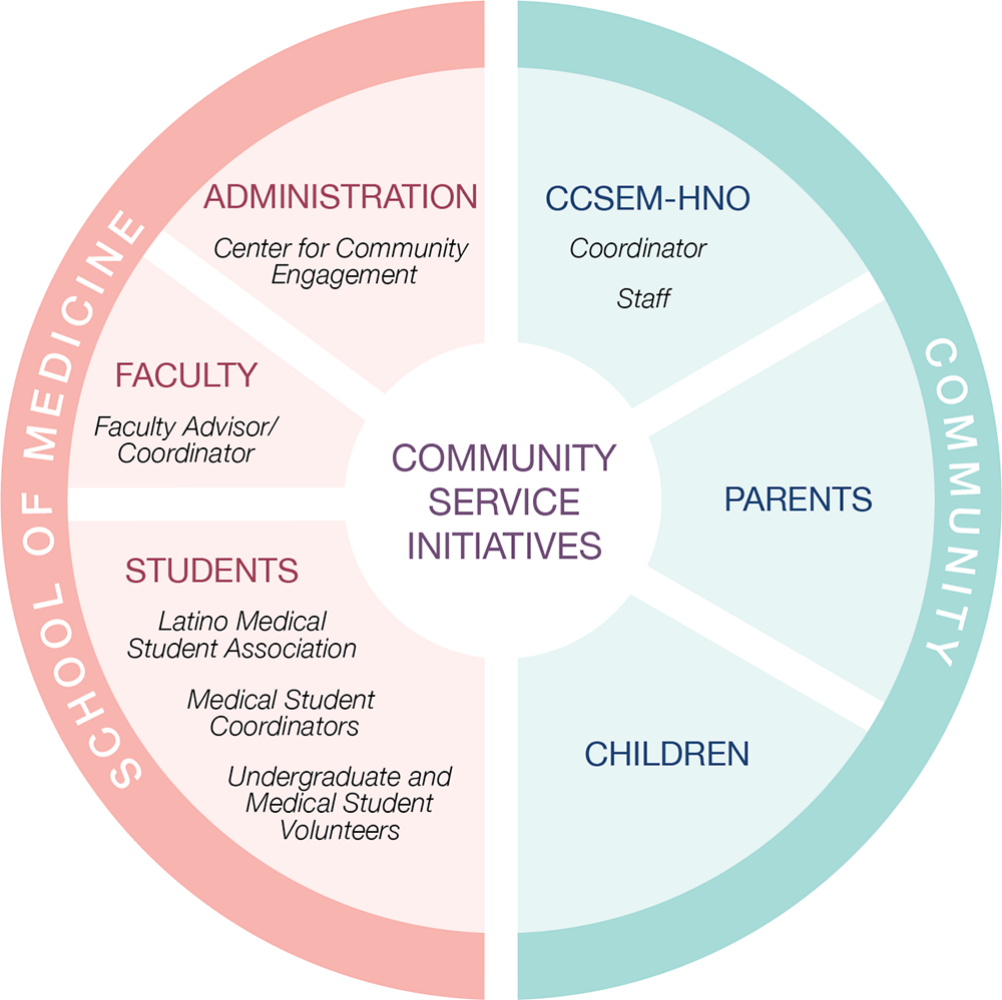
Building a community service partnership between the community and a school of medicine. CCSEM-HNO and OUWB worked collectively to provide human and financial resources for our community service initiative. Key personnel included the CCSEM-HNO coordinator, the medical school faculty advisor, and the medical student co-coordinators, with sponsorship by the Latino Medical Student Association chapter and the Center for Community Engagement at OUWB.

As described in Figure 1, the 4 year-long partnership between OUWB and CCSEM-HNO streamlined a very collaborative planning process to safeguard the program’s continuity. The strength of our partnership provided a dependable line of communication to efficiently switch gears from in-person to online sessions. The trust that had been built over many years allowed us to come to meetings and spend less time re-establishing each other’s roles, responsibilities, and capabilities. It became an opportunity to devote the vast majority of our time to troubleshoot potential issues (e.g., enrollment, packaging and distribution of program materials, unreliable WiFi, children’s limited experience with technology) and follow through with proposed solutions. Furthermore, our established partnership provided a proven ability to properly identify and direct resources from the medical school and the community.

### Leadership Structure

One major challenge in modifying the program in light of the pandemic was the time investment needed for decision-making, planning, and the growing number of additional responsibilities to successfully transition to a virtual model. We argue that, in addition to a strong, established partnership, a student-centered leadership structure is essential to effectively sustain a community service program during a public health crisis. In our experience, shaping our program to be student-driven and supported by a student association (the Latino Medical Student Association) became a strong attribute to the sustainability of the program. Our leadership structure consisted of four players—a community coordinator, a medical school faculty advisor, and medical student co-coordinators (Figure 1). Unlike the medical student coordinators, the community coordinator and the medical school faculty advisor had commitments to their institutions that were evolving heavily during the pandemic. As a result, the medical student coordinators, who generally had more time and flexibility within their schedules, assumed strategic advocacy roles to respond to the challenges that arose during the transition period. Three of the most labor-intensive outcomes of our student-centered leadership structure included redesigning the educational sessions, creating an adapted version of the instructor’s manual,^1^ and coordinating the structural logistics required to move from face-to-face to virtual sessions.

### Structural Logistics

The structural logistics, or the detailed coordination of resources, personnel, and online classroom design, became especially important during the transition. Three areas of concern that generated the most discussion during the planning phase included access to technology, training in technology, and classroom size and student-teacher ratio.

First, it was important to acknowledge that online programs rely on technology, and gaps in computer access and reliable internet connection continue to exist for Hispanic/Latino individuals (Perrin & Turner, 2019). A significant obstacle was student access to the technology needed to participate in the program. It was imperative for both the students and the instructors to have reliable access to encourage strong social interactions and verbal/nonverbal communication. Although we ensured that all students had access to a laptop, tablet, or phone with audio and video capabilities, some of them were siblings and shared one device. This scenario could have subsequently led to distractions and inhibited independent learning and problem-solving. However, we made certain to give each student their own program materials, thus allowing them to complete the art projects and interactive activities individually.

Second, in addition to access to technology, we prioritized proper training in technology for all personnel involved in the program. We conducted trial sessions among the students and program leaders on two viable video communication services (Zoom, Google Meet), not only to identify the best platform for official use but also to ensure the proper functioning of everyone’s cameras, microphones, and keyboards and observe the general dynamics of online learning.^2^ We concluded that Zoom was the best choice, given its reputation as reliable, widely used in K-12 education, and easy for school-age children to access. Regardless of its user-friendly interface, we implemented training sessions among the community coordinator, community staff members, and the medical student coordinators to become acquainted with the platform’s different features and adjust computer settings appropriately.

Third, we faced a necessary challenge of optimizing the classroom size and student-teacher ratio to accommodate some of the concerns of an online classroom (e.g., maintaining order, controlling noise volume, encouraging class participation). Therefore, we departed from the previous year’s large, 20-student classroom model (student-teacher ratio of 20:3) in favor of small groups of seven to eight students based on age (student-teacher ratio of 7–8:2). These structural changes helped to minimize audio-related overlap and disruptions that were evident in a trial session with around 30 students in one video conferencing room. The smaller classroom size also facilitated an intimate learning setting with more opportunities for personalized instruction, bonding, and formative feedback.

### Design of Educational Sessions

**Figure 2.**
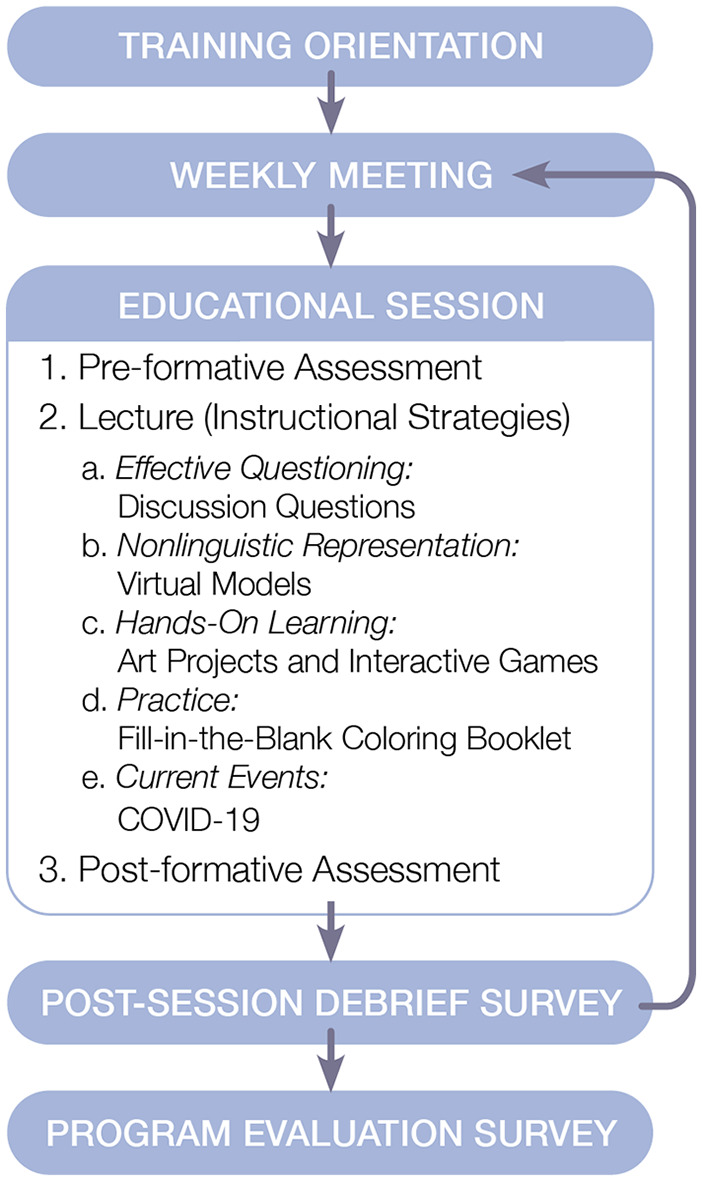
Logistical framework of our online program. The sequence of events for overall program delivery and the general format of the educational sessions are outlined.

One of the biggest hurdles was redesigning the educational sessions to reflect a sustainable, customizable model that provides future instructors more flexibility and reassurance. We aimed to create a model that allows most, if not all, elements of the learning format to be applicable not only during a public health crisis but also in the years to come. Figure 2 summarizes the general format of this year’s educational sessions contextualized within the timeline for volunteer training and overall program delivery. Below, we describe the most integral elements of the redesign with respect to the following categories: (a) special technological features, (b) instructional strategies, and (c) assessment and program evaluation.

#### Special technological features

In choosing the best technological platform for an online classroom, three special features—breakout rooms, screenshare, and the whiteboard—were considered. The breakout room feature allowed instructors to start the session as one large group (to share general instructions and announcements) and then seamlessly split into two smaller groups (for effective delivery of the teaching material and activities). The screenshare feature provided a means to project digital content (e.g., videos, games, models) and guide students’ attention to different areas of the screen with ease. The whiteboard feature became a communication channel to post general instructions and announcements at the start of class for quick reference. As previously mentioned, computer training and trial sessions were essential to troubleshooting technology-related problems and glitches.

#### Instructional strategies

In previous years with face-to-face instruction, the teaching philosophy was rooted in creating a fun, engaging experience for the children and incorporating multiple learning modalities (e.g., visuospatial learning, kinesthetic learning, think-pair-share technique, open discussion questions). In order to preserve this teaching philosophy, we focused on one particular attribute in becoming effective online teachers—“varying pedagogy”—and consequently diversified our teaching methods (Rose, 2018). This subsection will delve deeper into five specific instructional strategies used in this year’s online program (Community Training and Assistance Center, & Washoe County School District, 2015).

##### Effective questioning

In order to create engaging sessions, effective questioning was established in our instructional design. In particular, questions were written with the purpose to provide an exciting introduction to a given session, encourage students to brainstorm and respond at higher levels, and/or focus on important concepts.

##### Nonlinguistic representation

As a contrast to the language-based learning that tends to predominate within classroom instruction, nonlinguistic representation (e.g., physical models, diagrams, simulations) allows students to exercise the brain’s preference for image processing and storage (Pedersen & Finson, 2015). This year, a number of virtual anatomical models were used in place of manual anatomical models to provide a three-dimensional exploration of various systems and organs. These virtual models allowed us to more easily manipulate the models in front of a computer screen.

##### Hands-on learning

To encourage ‘learning by doing’ and address the restlessness that may come from long online sessions, we decided to consistently set aside structured time for arts and crafts activities and interactive games. Our lesson plans included at least one hands-on art project and one interactive game to reinforce concepts through fun, low-stress activities. For example, in one of our sessions, we invited students to create lung models (with the program materials provided ahead of time) and participate in a small experiment to understand the mechanics of breathing.

##### Practice

This learning tool allows students to review key ideas, apply learned concepts in a novel way, and refine their understanding through practice exercises. We extended opportunities for active learning and recall through a “labeling and coloring” booklet (which was printed and distributed to the students) to reinforce major anatomical structures and their spatial relationships.

##### Current events

The strategy of tying concepts to current events provides students the opportunity to understand and appreciate the present-day relevance of the information. For our program, every lesson plan included application exercises centered around COVID-19 to provide exposure to some of the distinguishing relationships between COVID-19 and human anatomy, with a secondary aim to help students feel more knowledgeable about the COVID-19 pandemic. For example, in one session, we explored the relative size and shape of the COVID-19 virus to grasp not only the visual limitations of the human eye but also the public health dangers of not being able to see the virus. It is also important to note that, in acknowledging the emotional impact that a public health crisis can have on children, we remained mindful of the sensitivity of the subject matter and kept explanations age-appropriate (National Association of School Psychologists, 2020).

#### Assessment and program evaluation

Both performance and program assessments are of vital importance to program sustainability. Although community service programs do not typically include assessments, we included pre- and post-assessments within each session to assess the effectiveness and age appropriateness of each lesson plan. We also conducted a program evaluation using student- and instructor-specific satisfaction surveys to identify areas of improvement and ensure that the program continually evolves. Although most of this year’s program was online, the assessments and program evaluations were completed on paper, as advised by the community coordinator, to avoid possible technological issues.

## Further Considerations

The immediate and more long-term impacts of COVID-19 on community service and education have yet to be evaluated. Considerable uncertainties persist for academia-sponsored community service initiatives in the U.S., especially with respect to the Latinx youth population. There will likely be challenges surrounding longevity, the availability of resources and funding, the future of education design (e.g., online, in-person, hybrid), the downstream effects of disrupted learning, and more (Kim, 2020). In light of the obstacles already identified by other countries, we are particularly concerned about the U.S. response to more equitable digital infrastructures, student ability to conduct self-directed learning, quality assurance for online learning, and providing social and emotional support to students (Zhu & Liu, 2020). Additionally, the absence of face-to-face interaction could impair educational competencies for medical students, including cultural competence, community service learning, cultural humility, emotional intelligence, and empathy (LCME, 2018).

In the meantime, we believe that attention should be directed to viable solutions that support and protect existing community service programs in the event of a large-scale disaster. One approach is to formulate a disaster plan between the community leaders and the academic institutions. Some components of the disaster plan could include clear, electronic documentation of all programming, blueprints, and guidelines as well as a list of all resources, potential grants, and funding options. In doing so, community service programs, especially those working with vulnerable populations and/or with limited resources, can take a proactive stance and be as prepared as possible for any crisis.

## Conclusion

We have described the challenges and opportunities of sustaining an established community service program geared toward the local Latinx youth population during the COVID-19 pandemic. Many aspects of the existing program required special consideration during the transition from in-person to virtual sessions, but the design and planning process allowed us to strengthen our partnership with community leaders, explore innovative teaching strategies, and ultimately aim for a sustainable model. Thus, our experience with developing an online community service model directly with the surrounding community could serve as a framework for others to improve the sustainability of their programs and effectively navigate uncertain times.

## Notes

1. Instructor’s manual is available upon request.

2. All online sessions involving the children required parental approval.
